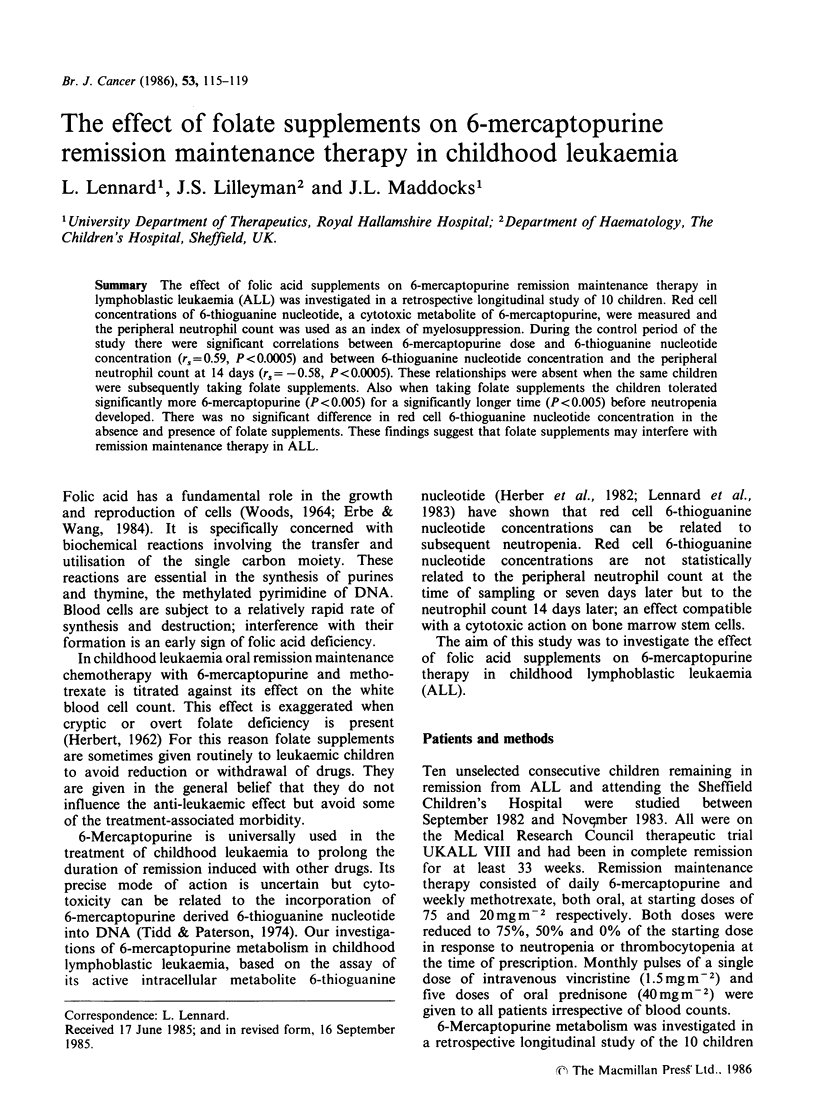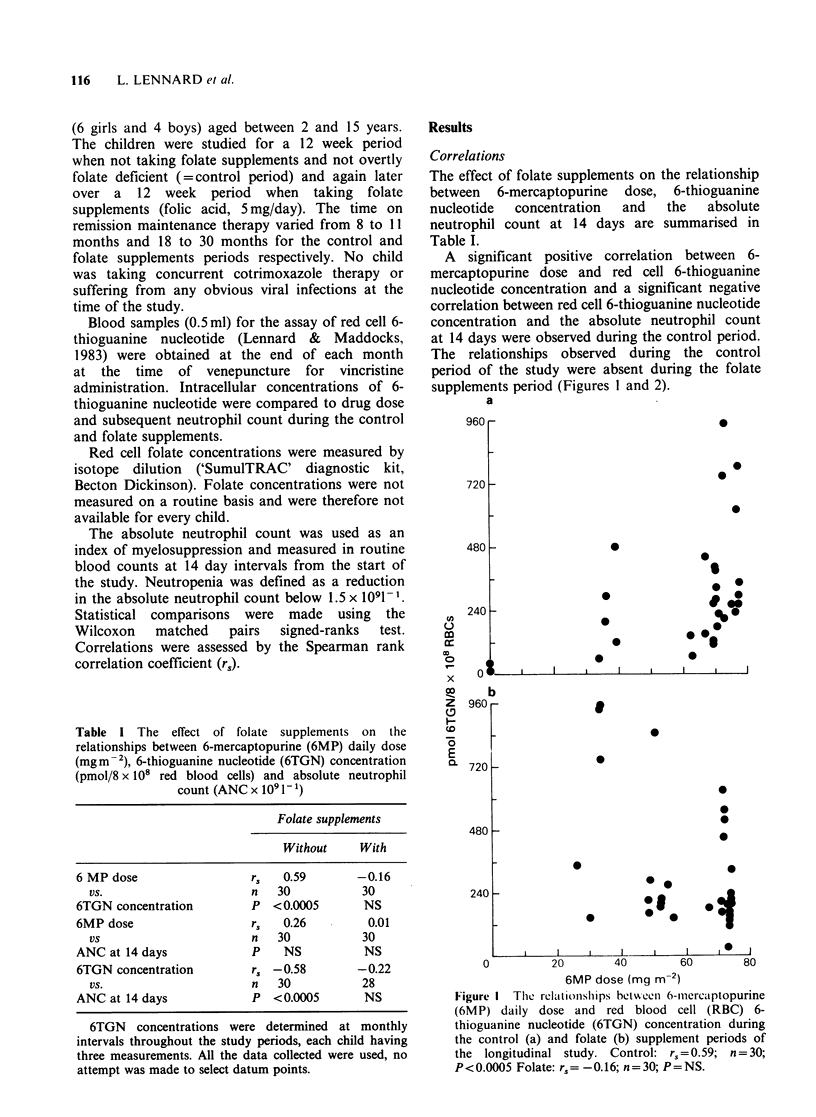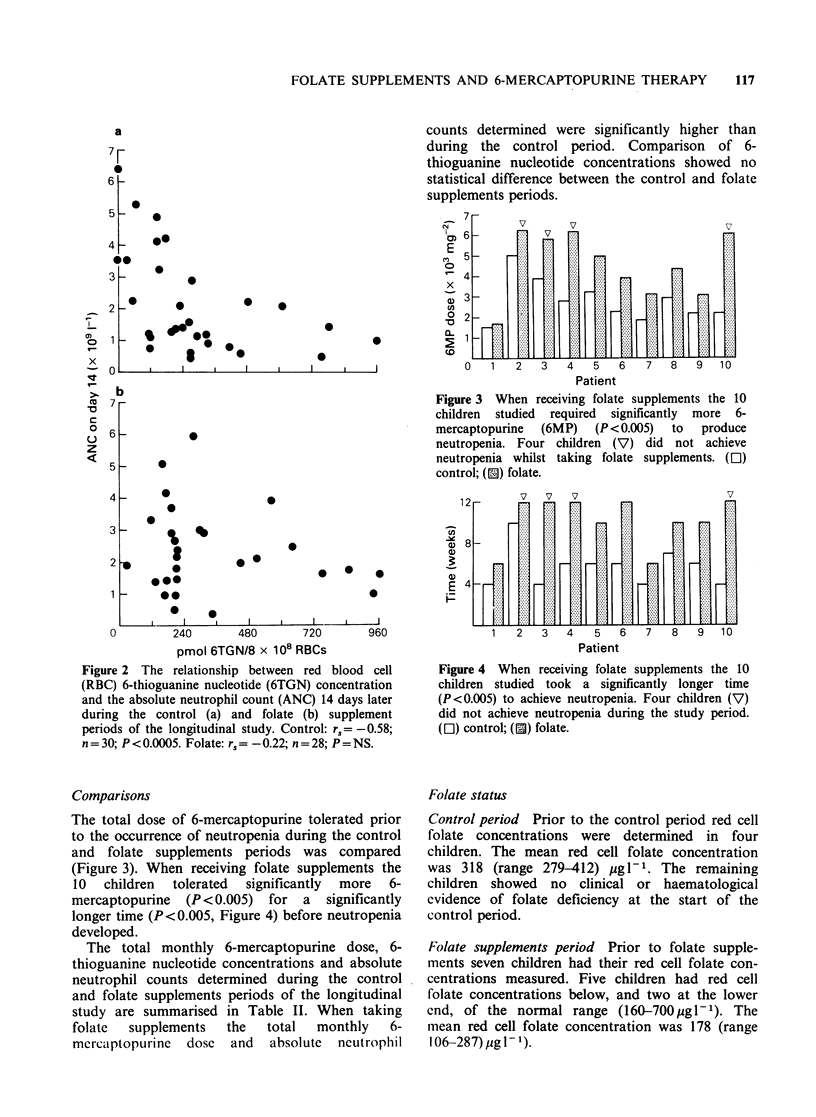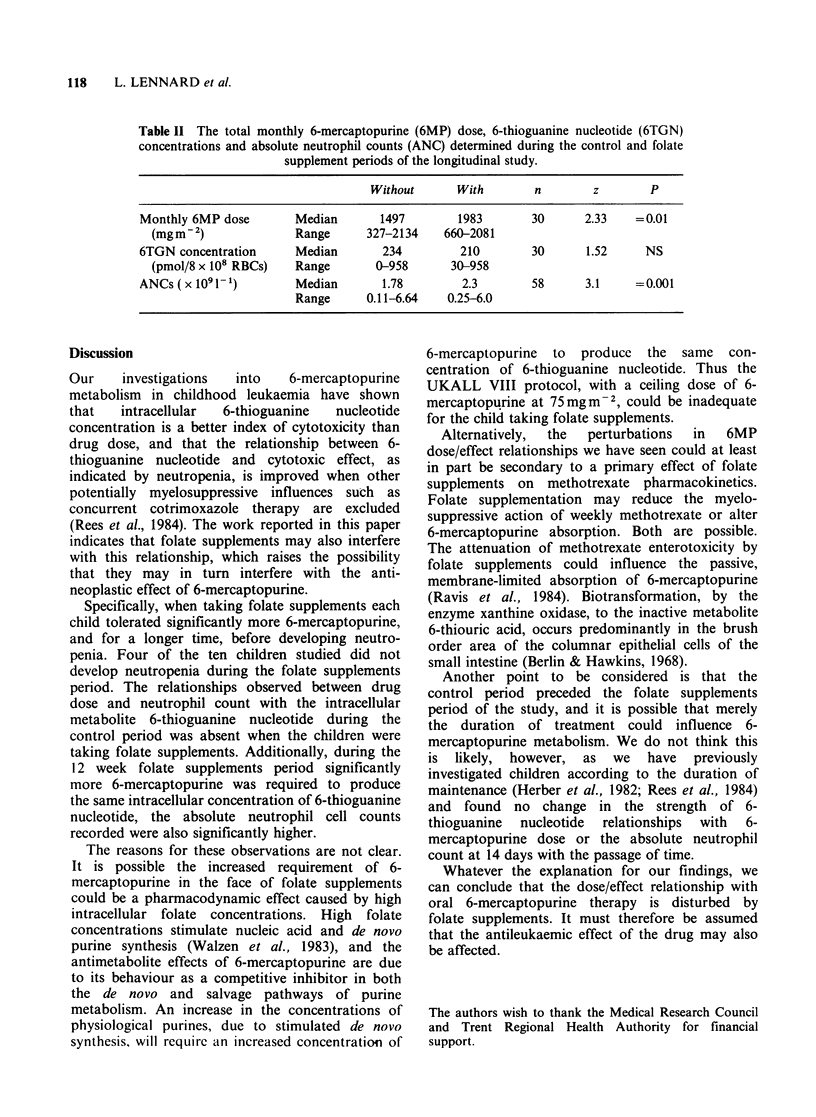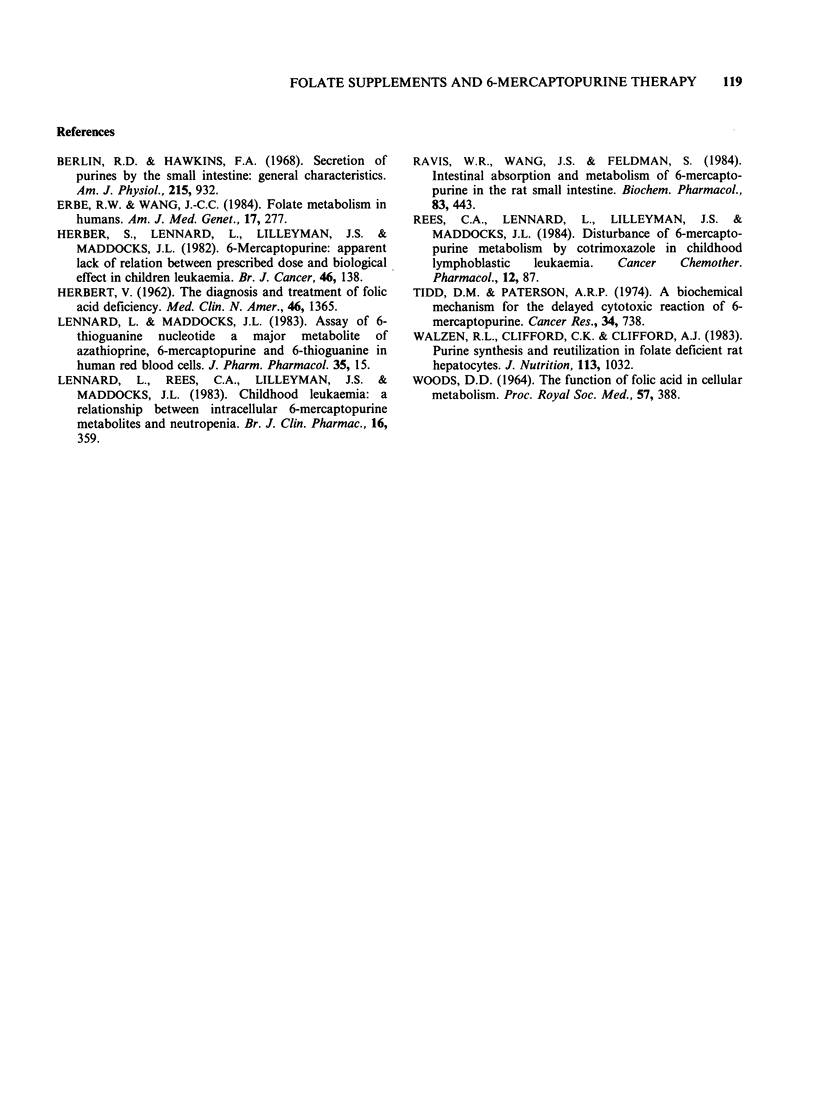# The effect of folate supplements on 6-mercaptopurine remission maintenance therapy in childhood leukaemia.

**DOI:** 10.1038/bjc.1986.16

**Published:** 1986-01

**Authors:** L. Lennard, J. S. Lilleyman, J. L. Maddocks

## Abstract

The effect of folic acid supplements on 6-mercaptopurine remission maintenance therapy in lymphoblastic leukaemia (ALL) was investigated in a retrospective longitudinal study of 10 children. Red cell concentrations of 6-thioguanine nucleotide, a cytotoxic metabolite of 6-mercaptopurine, were measured and the peripheral neutrophil count was used as an index of myelosuppression. During the control period of the study there were significant correlations between 6-mercaptopurine dose and 6-thioguanine nucleotide concentration (rs = 0.59, P less than 0.0005) and between 6-thioguanine nucleotide concentration and the peripheral neutrophil count at 14 days (rs = 0.58, P less than 0.0005). These relationships were absent when the same children were subsequently taking folate supplements. Also when taking folate supplements the children tolerated significantly more 6-mercaptopurine (P less than 0.005) for a significantly longer time (P less than 0.005) before neutropenia developed. There was no significant difference in red cell 6-thioguanine nucleotide concentration in the absence and presence of folate supplements. These findings suggest that folate supplements may interfere with remission maintenance therapy in ALL.


*Br. J. Cancer* (1986), **53**, 115–119

### The effect of folate supplements on 6-mercaptopurine remission maintenance therapy in childhood leukaemia

L. Lennard[1], J.S. Lilleyman[2] and J.L. Maddocks[1]

[1] *University Department of Therapeutics, Royal Hallamshire Hospital;* [2] *Department of Haematology, The Children's Hospital, Sheffield, UK.*

**Summary** The effect of folic acid supplements on 6-mercaptopurine remission maintenance therapy in lymphoblastic leukaemia (ALL) was investigated in a retrospective longitudinal study of 10 children. Red cell concentrations of 6-thioguanine nucleotide, a cytotoxic metabolite of 6-mercaptopurine, were measured and the peripheral neutrophil count was used as an index of myelosuppression. During the control period of the study there were significant correlations between 6-mercaptopurine dose and 6-thioguanine nucleotide concentration ($r_s = 0.59$, $P < 0.0005$) and between 6-thioguanine nucleotide concentration and the peripheral neutrophil count at 14 days ($r_s = -0.58$, $P < 0.0005$). These relationships were absent when the same children were subsequently taking folate supplements. Also when taking folate supplements the children tolerated significantly more 6-mercaptopurine ($P < 0.005$) for a significantly longer time ($P < 0.005$) before neutropenia developed. There was no significant difference in red cell 6-thioguanine nucleotide concentration in the absence and presence of folate supplements. These findings suggest that folate supplements may interfere with remission maintenance therapy in ALL.

Folic acid has a fundamental role in the growth and reproduction of cells (Woods, 1964; Erbe & Wang, 1984). It is specifically concerned with biochemical reactions involving the transfer and utilisation of the single carbon moiety. These reactions are essential in the synthesis of purines and thymine, the methylated pyrimidine of DNA. Blood cells are subject to a relatively rapid rate of synthesis and destruction; interference with their formation is an early sign of folic acid deficiency.

In childhood leukaemia oral remission maintenance chemotherapy with 6-mercaptopurine and methotrexate is titrated against its effect on the white blood cell count. This effect is exaggerated when cryptic or overt folate deficiency is present (Herbert, 1962) For this reason folate supplements are sometimes given routinely to leukaemic children to avoid reduction or withdrawal of drugs. They are given in the general belief that they do not influence the anti-leukaemic effect but avoid some of the treatment-associated morbidity.

6-Mercaptopurine is universally used in the treatment of childhood leukaemia to prolong the duration of remission induced with other drugs. Its precise mode of action is uncertain but cytotoxicity can be related to the incorporation of 6-mercaptopurine derived 6-thioguanine nucleotide into DNA (Tidd & Paterson, 1974). Our investigations of 6-mercaptopurine metabolism in childhood lymphoblastic leukaemia, based on the assay of its active intracellular metabolite 6-thioguanine nucleotide (Herber *et al.*, 1982; Lennard *et al.*, 1983) have shown that red cell 6-thioguanine nucleotide concentrations can be related to subsequent neutropenia. Red cell 6-thioguanine nucleotide concentrations are not statistically related to the peripheral neutrophil count at the time of sampling or seven days later but to the neutrophil count 14 days later; an effect compatible with a cytotoxic action on bone marrow stem cells.

The aim of this study was to investigate the effect of folic acid supplements on 6-mercaptopurine therapy in childhood lymphoblastic leukaemia (ALL).

### Patients and methods

Ten unselected consecutive children remaining in remission from ALL and attending the Sheffield Children's Hospital were studied between September 1982 and November 1983. All were on the Medical Research Council therapeutic trial UKALL VIII and had been in complete remission for at least 33 weeks. Remission maintenance therapy consisted of daily 6-mercaptopurine and weekly methotrexate, both oral, at starting doses of 75 and 20 mg m$^{-2}$ respectively. Both doses were reduced to 75%, 50% and 0% of the starting dose in response to neutropenia or thrombocytopenia at the time of prescription. Monthly pulses of a single dose of intravenous vincristine (1.5 mg m$^{-2}$) and five doses of oral prednisone (40 mg m$^{-2}$) were given to all patients irrespective of blood counts.

6-Mercaptopurine metabolism was investigated in a retrospective longitudinal study of the 10 children

Correspondence: L. Lennard.
Received 17 June 1985; and in revised form, 16 September 1985.

© The Macmillan Press Ltd., 1986

(6 girls and 4 boys) aged between 2 and 15 years. The children were studied for a 12 week period when not taking folate supplements and not overtly folate deficient (=control period) and again later over a 12 week period when taking folate supplements (folic acid, 5 mg/day). The time on remission maintenance therapy varied from 8 to 11 months and 18 to 30 months for the control and folate supplements periods respectively. No child was taking concurrent cotrimoxazole therapy or suffering from any obvious viral infections at the time of the study.

Blood samples (0.5 ml) for the assay of red cell 6-thioguanine nucleotide (Lennard & Maddocks, 1983) were obtained at the end of each month at the time of venepuncture for vincristine administration. Intracellular concentrations of 6-thioguanine nucleotide were compared to drug dose and subsequent neutrophil count during the control and folate supplements.

Red cell folate concentrations were measured by isotope dilution ('SumulTRAC' diagnostic kit, Becton Dickinson). Folate concentrations were not measured on a routine basis and were therefore not available for every child.

The absolute neutrophil count was used as an index of myelosuppression and measured in routine blood counts at 14 day intervals from the start of the study. Neutropenia was defined as a reduction in the absolute neutrophil count below $1.5 \times 10^9 l^{-1}$. Statistical comparisons were made using the Wilcoxon matched pairs signed-ranks test. Correlations were assessed by the Spearman rank correlation coefficient ($r_s$).

**Table 1** The effect of folate supplements on the relationships between 6-mercaptopurine (6MP) daily dose (mg m$^{-2}$), 6-thioguanine nucleotide (6TGN) concentration (pmol/8 × $10^8$ red blood cells) and absolute neutrophil count (ANC × $10^9 l^{-1}$)

| | | *Folate supplements* | |
|---|---|---|---|
| | | *Without* | *With* |
| 6 MP dose | $r_s$ | 0.59 | −0.16 |
| *vs.* | $n$ | 30 | 30 |
| 6TGN concentration | $P$ | <0.0005 | NS |
| 6MP dose | $r_s$ | 0.26 | 0.01 |
| *vs* | $n$ | 30 | 30 |
| ANC at 14 days | $P$ | NS | NS |
| 6TGN concentration | $r_s$ | −0.58 | −0.22 |
| *vs.* | $n$ | 30 | 28 |
| ANC at 14 days | $P$ | <0.0005 | NS |

6TGN concentrations were determined at monthly intervals throughout the study periods, each child having three measurements. All the data collected were used, no attempt was made to select datum points.

### Results

### *Correlations*

The effect of folate supplements on the relationship between 6-mercaptopurine dose, 6-thioguanine nucleotide concentration and the absolute neutrophil count at 14 days are summarised in Table I.

A significant positive correlation between 6-mercaptopurine dose and red cell 6-thioguanine nucleotide concentration and a significant negative correlation between red cell 6-thioguanine nucleotide concentration and the absolute neutrophil count at 14 days were observed during the control period. The relationships observed during the control period of the study were absent during the folate supplements period (Figures 1 and 2).

**Figure 1** The relationships between 6-mercaptopurine (6MP) daily dose and red blood cell (RBC) 6-thioguanine nucleotide (6TGN) concentration during the control (a) and folate (b) supplement periods of the longitudinal study. Control: $r_s = 0.59$; $n = 30$; $P < 0.0005$ Folate: $r_s = -0.16$; $n = 30$; $P = $NS.

**Figure 2** The relationship between red blood cell (RBC) 6-thioguanine nucleotide (6TGN) concentration and the absolute neutrophil count (ANC) 14 days later during the control (a) and folate (b) supplement periods of the longitudinal study. Control: $r_s = -0.58$; $n=30$; $P<0.0005$. Folate: $r_s = -0.22$; $n=28$; $P=$NS.

counts determined were significantly higher than during the control period. Comparison of 6-thioguanine nucleotide concentrations showed no statistical difference between the control and folate supplements periods.

**Figure 3** When receiving folate supplements the 10 children studied required significantly more 6-mercaptopurine (6MP) ($P<0.005$) to produce neutropenia. Four children (▽) did not achieve neutropenia whilst taking folate supplements. (□) control; (▨) folate.

**Figure 4** When receiving folate supplements the 10 children studied took a significantly longer time ($P<0.005$) to achieve neutropenia. Four children (▽) did not achieve neutropenia during the study period. (□) control; (▨) folate.

*Comparisons*

The total dose of 6-mercaptopurine tolerated prior to the occurrence of neutropenia during the control and folate supplements periods was compared (Figure 3). When receiving folate supplements the 10 children tolerated significantly more 6-mercaptopurine ($P<0.005$) for a significantly longer time ($P<0.005$, Figure 4) before neutropenia developed.

The total monthly 6-mercaptopurine dose, 6-thioguanine nucleotide concentrations and absolute neutrophil counts determined during the control and folate supplements periods of the longitudinal study are summarised in Table II. When taking folate supplements the total monthly 6-mercaptopurine dose and absolute neutrophil

*Folate status*

*Control period* Prior to the control period red cell folate concentrations were determined in four children. The mean red cell folate concentration was 318 (range 279–412) $\mu g\,l^{-1}$. The remaining children showed no clinical or haematological evidence of folate deficiency at the start of the control period.

*Folate supplements period* Prior to folate supplements seven children had their red cell folate concentrations measured. Five children had red cell folate concentrations below, and two at the lower end, of the normal range (160–700 $\mu g\,l^{-1}$). The mean red cell folate concentration was 178 (range 106–287) $\mu g\,l^{-1}$).

**Table II** The total monthly 6-mercaptopurine (6MP) dose, 6-thioguanine nucleotide (6TGN) concentrations and absolute neutrophil counts (ANC) determined during the control and folate supplement periods of the longitudinal study.

| | | *Without* | *With* | *n* | *z* | *P* |
|---|---|---|---|---|---|---|
| Monthly 6MP dose ($mg\,m^{-2}$) | Median | 1497 | 1983 | 30 | 2.33 | =0.01 |
| | Range | 327–2134 | 660–2081 | | | |
| 6TGN concentration ($pmol/8 \times 10^8$ RBCs) | Median | 234 | 210 | 30 | 1.52 | NS |
| | Range | 0–958 | 30–958 | | | |
| ANCs ($\times 10^9\,l^{-1}$) | Median | 1.78 | 2.3 | 58 | 3.1 | =0.001 |
| | Range | 0.11–6.64 | 0.25–6.0 | | | |

### Discussion

Our investigations into 6-mercaptopurine metabolism in childhood leukaemia have shown that intracellular 6-thioguanine nucleotide concentration is a better index of cytotoxicity than drug dose, and that the relationship between 6-thioguanine nucleotide and cytotoxic effect, as indicated by neutropenia, is improved when other potentially myelosuppressive influences such as concurrent cotrimoxazole therapy are excluded (Rees *et al.*, 1984). The work reported in this paper indicates that folate supplements may also interfere with this relationship, which raises the possibility that they may in turn interfere with the anti-neoplastic effect of 6-mercaptopurine.

Specifically, when taking folate supplements each child tolerated significantly more 6-mercaptopurine, and for a longer time, before developing neutropenia. Four of the ten children studied did not develop neutropenia during the folate supplements period. The relationships observed between drug dose and neutrophil count with the intracellular metabolite 6-thioguanine nucleotide during the control period was absent when the children were taking folate supplements. Additionally, during the 12 week folate supplements period significantly more 6-mercaptopurine was required to produce the same intracellular concentration of 6-thioguanine nucleotide, the absolute neutrophil cell counts recorded were also significantly higher.

The reasons for these observations are not clear. It is possible the increased requirement of 6-mercaptopurine in the face of folate supplements could be a pharmacodynamic effect caused by high intracellular folate concentrations. High folate concentrations stimulate nucleic acid and *de novo* purine synthesis (Walzen *et al.*, 1983), and the antimetabolite effects of 6-mercaptopurine are due to its behaviour as a competitive inhibitor in both the *de novo* and salvage pathways of purine metabolism. An increase in the concentrations of physiological purines, due to stimulated *de novo* synthesis, will require an increased concentration of 6-mercaptopurine to produce the same concentration of 6-thioguanine nucleotide. Thus the UKALL VIII protocol, with a ceiling dose of 6-mercaptopurine at $75\,mg\,m^{-2}$, could be inadequate for the child taking folate supplements.

Alternatively, the perturbations in 6MP dose/effect relationships we have seen could at least in part be secondary to a primary effect of folate supplements on methotrexate pharmacokinetics. Folate supplementation may reduce the myelosuppressive action of weekly methotrexate or alter 6-mercaptopurine absorption. Both are possible. The attenuation of methotrexate enterotoxicity by folate supplements could influence the passive, membrane-limited absorption of 6-mercaptopurine (Ravis *et al.*, 1984). Biotransformation, by the enzyme xanthine oxidase, to the inactive metabolite 6-thiouric acid, occurs predominantly in the brush order area of the columnar epithelial cells of the small intestine (Berlin & Hawkins, 1968).

Another point to be considered is that the control period preceded the folate supplements period of the study, and it is possible that merely the duration of treatment could influence 6-mercaptopurine metabolism. We do not think this is likely, however, as we have previously investigated children according to the duration of maintenance (Herber *et al.*, 1982; Rees *et al.*, 1984) and found no change in the strength of 6-thioguanine nucleotide relationships with 6-mercaptopurine dose or the absolute neutrophil count at 14 days with the passage of time.

Whatever the explanation for our findings, we can conclude that the dose/effect relationship with oral 6-mercaptopurine therapy is disturbed by folate supplements. It must therefore be assumed that the antileukaemic effect of the drug may also be affected.

The authors wish to thank the Medical Research Council and Trent Regional Health Authority for financial support.

### References

BERLIN, R.D. & HAWKINS, F.A. (1968). Secretion of purines by the small intestine: general characteristics. *Am. J. Physiol.*, **215**, 932.

ERBE, R.W. & WANG, J.-C.C. (1984). Folate metabolism in humans. *Am. J. Med. Genet.*, **17**, 277.

HERBER, S., LENNARD, L., LILLEYMAN, J.S. & MADDOCKS, J.L. (1982). 6-Mercaptopurine: apparent lack of relation between prescribed dose and biological effect in children leukaemia. *Br. J. Cancer*, **46**, 138.

HERBERT, V. (1962). The diagnosis and treatment of folic acid deficiency. *Med. Clin. N. Amer.*, **46**, 1365.

LENNARD, L. & MADDOCKS, J.L. (1983). Assay of 6-thioguanine nucleotide a major metabolite of azathioprine, 6-mercaptopurine and 6-thioguanine in human red blood cells. *J. Pharm. Pharmacol.* **35**, 15.

LENNARD, L., REES, C.A., LILLEYMAN, J.S. & MADDOCKS, J.L. (1983). Childhood leukaemia: a relationship between intracellular 6-mercaptopurine metabolites and neutropenia. *Br. J. Clin. Pharmac.*, **16**, 359.

RAVIS, W.R., WANG, J.S. & FELDMAN, S. (1984). Intestinal absorption and metabolism of 6-mercaptopurine in the rat small intestine. *Biochem. Pharmacol.*, **83**, 443.

REES, C.A., LENNARD, L., LILLEYMAN, J.S. & MADDOCKS, J.L. (1984). Disturbance of 6-mercaptopurine metabolism by cotrimoxazole in childhood lymphoblastic leukaemia. *Cancer Chemother. Pharmacol.*, **12**, 87.

TIDD, D.M. & PATERSON, A.R.P. (1974). A biochemical mechanism for the delayed cytotoxic reaction of 6-mercaptopurine. *Cancer Res.*, **34**, 738.

WALZEN, R.L., CLIFFORD, C.K. & CLIFFORD, A.J. (1983). Purine synthesis and reutilization in folate deficient rat hepatocytes. *J. Nutrition*, **113**, 1032.

WOODS, D.D. (1964). The function of folic acid in cellular metabolism. *Proc. Royal Soc. Med.*, **57**, 388.